# Crossroad of motor neuron disease and dementia: Insights from TDP-43 RNA-binding deficiency

**DOI:** 10.4103/NRR.NRR-D-25-01285

**Published:** 2026-01-27

**Authors:** Molly Magarotto, Han-Jou Chen

**Affiliations:** York Biomedical Research Institute, Department of Biology, University of York, Wentworth Way, Yorkshire, UK

With our ever-increasing aging population, age-related health issues are becoming one of the greatest sociological and economic pressures on society. This is particularly the case for neurodegenerative diseases that are estimated to affect over 1 in 3 people globally without effective treatment so far. Neurodegenerative disorders are broadly characterized by the gradual deterioration and death of neurons in the brain and/or spinal cord that leads to irreversible damage to the nervous system. Depending on the types of neurons and brain region affected, neurodegenerative diseases manifest as motor disruptions such as Huntington’s disease, amyotrophic lateral sclerosis (ALS), and Parkinson’s disease to dementias such as Alzheimer’s disease and frontotemporal dementia (FTD). All neurodegenerative diseases are associated with aberrant protein accumulation, which contributes to neuron toxicity and disease development. However, the mechanisms involved in the selective vulnerability for different types of neurons driving various clinical displays remain unclear.

ALS, despite primarily being recognized as a motor neuron disease, is on a spectrum with FTD, and therefore provides a great opportunity to investigate the mechanisms and pathology underlying the presentation of motor and/or cognitive symptoms. ALS is predominantly characterized by the progressive loss of the upper motor neurons of the cortex and brain stem, and the lower motor neurons of the spinal cord, with common symptoms including muscle atrophy and weakness. Bulbar dysfunction and respiratory deficits are the most common causes of mortality, occurring 2–5 years post-diagnosis. However, approximately 15% of ALS patients are diagnosed with concomitant FTD, and up to 50% of ALS patients show cognitive function impairment without a full dementia diagnosis. Furthermore, ALS and FTD share many genetic and pathological features, indicating there are likely converging mechanisms underlying the development of these diseases.

**Figure 1:**
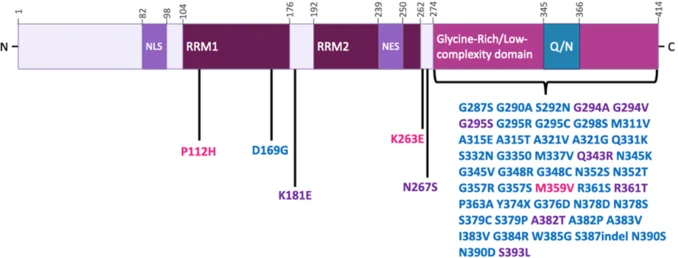
ALS/FTD-associated mutations to *TARDBP*. Blue: Mutations exclusive to ALS; Pink: mutations exclusive to FTD; Purple: mutations linked to both ALS and FTD. Numbering indicates amino acid positions of TDP-43 domains. TDP-43 structure adapted from Chen et al. (2019). ALS: Amyotrophic lateral sclerosis; FTD: frontotemporal dementia; NES: nuclear export signal; NLS: nuclear localization signal; RRM1/RRM2: RNA-recognition motifs; TDP-43: TAR DNA-binding protein 43.

A key disease-associated protein is TAR DNA-binding protein 43 (TDP-43), the pathology of which is observed in 97% of ALS, 50% of FTD and 57% of AD. It is also prominent in limbic-predominant age-related TDP-43 encephalopathy (LATE), and in some cases of Parkinson’s disease and Huntington’s disease. TDP-43 contains a nuclear localization signal aiding nucleocytoplasmic transport, two RNA-recognition motifs (RRMs) that are responsible for RNA-binding and an aggregation-prone low complexity domain C-terminal. In disease, TDP-43 mislocalizes to the cytoplasm where it forms hyperphosphorylated and ubiquitinated aggregates, referred to as TDP-43 proteinopathy. There are around 50 causative or disease-modifying mutations to the *TARDBP* gene associated with familial ALS, with the majority situated in the C-terminal (**Figure 1**). A few rare mutations are found in and around the RRMs, including *P112H*, *D169G*, and *K181E* situated around RRM1, and *K263E* and *N267S* situated adjacent to RRM2. Interestingly, all except D169G are associated with FTD, while K181E and N267S occur with ALS and FTD within the same families (**Table 1**). Furthermore, this link to FTD is correlated with the impact of the mutation on RNA-interactions as the ALS/FTD linked *K181E*, *K263E*, and *P112H* disrupt RNA interaction, while the ALS-associated *D169G* does not (Chen et al., 2019). Similarly, the vast majority of C-terminal mutations are ALS-associated, and while they can affect bound RNA specifics, they do not disrupt TDP-43 RNA-binding (Chen et al., 2019). Together, the genetic data seem to suggest that TDP-43 RNA-binding deficiency more readily contributes to the cognitive phenotype. Indeed, this hypothesis is supported by a recent transgenic mouse study where expression of the RNA-binding deficient acetylation-mimic (K145Q) TDP-43 causes cognitive rather than motor phenotypes, with a transcriptomic profile more similar to FTD than ALS (Necarsulmer et al., 2023). But how does RNA-binding deficient TDP-43 prompt these cognitive phenotypes?

**Table 1:** Disease-associated mutations surrounding TDP-43 RRMs

Mutation	Disease association	Affecting RNA-binding
*P112H*	FTD	Yes
*D169G*	ALS	No
*K181E*	FTD, ALS	Yes
*K263E*	FTD	Yes
*N267S*	FTD, ALS, Parkinson’s disease	No published impact on RNA-binding to date

ALS: Amyotrophic lateral sclerosis; FTD: frontotemporal dementia.

TDP-43 is a ubiquitously expressed protein that is aggregation prone. Despite TDP-43 pathology primarily observed in the motor cortex and spinal cord in typical ALS, it can be observed to spread to other brain areas, including the cortical and subcortical areas of the temporal and occipital lobes, and the striatum and thalamus, potentially even reaching the cerebellum in some cases (Jo et al., 2020). As ALS is an aggressive disease with average survival rates of 2–5 years post-diagnosis, it is possible that both motor and cortical neurons are indeed affected by TDP-43 toxicity, with patients succumbing to the prominent failure associated with motor function disruption before cognitive symptoms are fully developed. Therefore, the link of RNA-binding-deficient TDP-43 to FTD could implicate a slower and milder toxicity that allows the spread to cortical neurons and brain regions. Indeed, cell studies show that K181E-TDP-43 and K263E-TDP-43 caused minimal toxicity when expressed at endogenous levels (Magarotto et al., 2025). Furthermore, expression of non-disease linked engineered RNA-binding-deficient TDP-43 in *Drosophila* and mouse models resulted in mild neurodegeneration that did not result in noticeable motor dysfunction (Ihara et al., 2013).

One possible contributor to this delayed/milder toxicity is the impact of the RNA-binding-deficient mutations on TDP-43 redistribution. The hallmark of TDP-43 proteinopathy is its cytoplasmic translocation and aggregation. Indeed, several cell, *Drosophila* and mouse models have shown that cytoplasmic TDP-43 with and without aggregation causes severe cytotoxicity and motor function defects (Walker et al., 2015). ALS-associated C-terminal mutants Q331K and M337V have been shown to more readily translocate to the cytoplasm (Chou et al., 2018). However, RNA-binding-deficient TDP-43 preferentially forms aggregates in the nucleus (Chen et al., 2019), which has also been observed in patients carrying the FTD-related P112H and K263E mutations. These nuclear aggregates exhibit decreased mobility and increased stability, and rarely translocate to the cytoplasm (Chen et al., 2019). Therefore, we hypothesize that cells carrying RNA-binding-deficient TDP-43 may have a slower rate of TDP-43 cyto-accumulation due to the increased formation of stable nuclear aggregates, allowing for a prolonged build-up in toxicity and spread to other brain regions.

Another possible mechanism underlying the delayed toxicity is the compensatory response triggered by the heterozygous expression of RNA-binding-deficient TDP-43 that sustains the neurons at the early stage of the pathological assault. Heterozygous K181E-TDP expression at endogenous levels exhibits increased neuronal gene regulation and consequential neurite outgrowth in our CRISPR/Cas9-knock-in to SH-SY5Y cells (Magarotto et al., 2025). Indeed, increased neuronal growth is a documented result of cellular stress such as oxidative stress or in response to neuronal injury. It is likely that this compensatory response eventually reaches a tipping point, where neurons enter the typical degenerative and apoptotic pathways characteristic in end-stage disease. However, compensatory neuronal growth as an early disease stage pathological mechanism is not well characterized. Increased dendritic branching has been observed in compensation for neighboring neuronal death in mice models of ALS (Clark et al., 2016); however, more studies are needed that better characterize early disease mechanisms across ALS and FTD ahead of late-stage neurodegeneration.

Conversely, homozygous K181E-TDP causes a massive down-regulation of neuronal and synaptic genes, including key targets of TDP-43 such as *STMN2*, consequently affecting neuronal health and function (Magarotto et al., 2025). Similar can be observed in iPSC-derived cortical neurons with homozygous K263E-TDP-43 knock-in (Imaizumi et al., 2022). The homozygous expression of RNA-binding-deficient TDP-43 mostly recapitulate what is observed in TDP-43 knock-down studies, including the increased cryptic exon inclusion and the down regulation of key neuronal transcripts, such as *STMN2* (Mehta et al., 2023). The increased cryptic exons are also observed in post-mortem tissue of ALS, FTD, and AD patients (Mehta et al., 2023), indicating that TDP-43 loss of function is apparent in the end stage of the diseases. However, ALS/FTD-associated mutations express in an autosomal dominant inheritance pattern, and divergence between homozygous and heterozygous RNA-binding-deficient mutations argues against a simple loss-of-function mechanism in early disease, but suggests that the early disease pathogenesis is driven by a complex gain-of-function or altered functions that dictate the initial clinical phenotypes. These differences early in the disease may better explain the development of cognitive and/or motor phenotypes despite similar end-stage pathology.

To summarize, TDP-43 is a key pathological protein in ALS, FTD, LATE, and AD. TDP-43 dysfunction is widely studied and accepted as a critical contributor to neuronal toxicity. However, how it is linked to various disease manifestations remains to be elucidated. Recent studies by others and us on ALS/FTD-associated RNA-binding-deficient TDP-43 shed some light on the pathogenesis underlying the cognitive-motor phenotype spectrum. RNA-deficient mutations cause milder and delayed toxicity in neurons that could be attributed to their tendency to form nuclear aggregates that result in reduced cytoplasmic translocation, and/or their induction of stress responsive compensatory mechanisms. This reduced toxicity, in turn, could prolong disease manifestation, allowing for wider pathological spread to other neuronal subtypes and brain regions that are more readily associated with cognitive decline. A broader study is needed to test these hypotheses and provide better understanding of TDP-43-mediated pathogenesis.

*This work was supported by the Academy of Medical Science (AMS, with Wellcome Trust, BEIS, the British Heart Foundation and Diabetes UK) [SBF006\1088] to HJC; and the Biology departmental PhD studentship to MM.*
